# In Vivo Estimation of the Biological Effects of Endocrine Disruptors in Rabbits after Combined and Long-Term Exposure: Study Protocol

**DOI:** 10.3390/toxics10050246

**Published:** 2022-05-12

**Authors:** Vasiliki Karzi, Manolis N. Tzatzarakis, Athanasios Alegakis, Elena Vakonaki, Irene Fragkiadoulaki, Konstantinos Kaloudis, Christina Chalkiadaki, Paraskevi Apalaki, Maria Panagiotopoulou, Aikaterini Kalliantasi, Demetrios Kouretas, Anca Oana Docea, Daniela Calina, Aristidis Tsatsakis

**Affiliations:** 1 Center of Toxicology, Medicine School, University of Crete, 70013 Heraklion, Greece; chemstud.vas2010@gmail.com (V.K.); tzatzarakis@uoc.gr (M.N.T.); alegkaka@uoc.gr (A.A.); evakonaki@gmail.com (E.V.); eirinimbg@hotmail.gr (I.F.); kaloudis@uoc.gr (K.K.); christina4495@hotmail.com (C.C.); bio2278apalaki@gmail.com (P.A.); mpanagiotopoulou22@gmail.com (M.P.); kalliantash_k@hotmail.com (A.K.); 2 Department of Biochemistry and Biotechnology, University of Thessaly, Viopolis, Mezourlo, 41500 Larissa, Greece; dkouret@uth.gr; 3 Department of Clinical Pharmacy, University of Medicine and Pharmacy of Craiova, 200349 Craiova, Romania; 4 Department of Toxicology, University of Medicine and Pharmacy of Craiova, 200349 Craiova, Romania

**Keywords:** glyphosate, parabens, triclosan, di (2-ethylhexyl) phthalate, rabbits, combined exposure, chronic exposure

## Abstract

Recently, an increasing number of chemical compounds are being characterized as endocrine disruptors since they have been proven to interact with the endocrine system, which plays a crucial role in the maintenance of homeostasis. Glyphosate is the active substance of the herbicide Roundup^®^, bisphenol A (BPA) and di (2-ethylhexyl) phthalate (DEHP) are used as plasticizers, while triclosan (TCS), methyl (MePB), propyl (PrPB), and butyl (BuPB) parabens are used as antimicrobial agents and preservatives mainly in personal care products. Studies indicate that exposure to these substances can affect humans causing developmental problems and problems in the endocrine, reproductive, nervous, immune, and respiratory systems. Although there are copious studies related to these substances, there are few in vivo studies related to combined exposure to these endocrine disruptors. The aim of the present pilot study is the investigation and assessment of the above substances’ toxicity in rabbits after twelve months of exposure to glyphosate (both pure and commercial form) and to a mixture of all the above substances at subtoxic levels. The lack of data from the literature concerning rabbits’ exposure to these substances and the restrictions of the 3Rs Principle will result in a limited number of animals available for use (four animals per group, twenty animals in total).

## 1. Introduction

The constant exposure of the population to a variety of chemicals is a global problem and has been of particular concern to the scientific community. In particular, exposure to substances suspected of being endocrine disruptors (EDCs) has been studied by many researchers. The endocrine system is a system of glands that control the proper functioning and action of hormones. Endocrine disruptors are chemicals that can interact with the endocrine system—a system that plays an important role in maintaining the body′s homeostasis—usually causing adverse effects on the body. This disorder leads to developmental problems as well as problems in the reproductive, nervous, respiratory, and immune systems [1].

Existing in vivo and in vitro studies mainly investigate the action and potential effects of a single substance. In addition, guidelines usually indicate whether or not a single substance is associated with endocrine action. However, people are exposed to a variety of chemicals at a daily rate, which raises concerns about the effects that the possible synergistic action that all of these substances may have on an organism [2]. Over the last five years, researchers have focused on determining the effects of real-life exposure on human organisms, the so-called real life risk simulation (RLRS), which proposes new approaches to exposure scenarios [3,4].

This study aims to investigate the daily effects that the exposure to chemical substances has on people, as these effects have several applications. In other words, our goal is to simulate the effects of chemical compounds in real life. In particular, the synergistic activity of glyphosate, bisphenol A (BPA), parabens (PBs), triclosan (TCS), and bis (2-ethylhexyl) phthalate (DEHP) in rabbits after twelve months of combined exposure will be evaluated. The toxicity of the above substances will be assessed both at the molecular and histological bases. At the same time, the effects after twelve months of exposure to the pure form of glyphosate will be compared with its commercial form (Roundup^®^ herbicide).

Glyphosate

Glyphosate (N-(phosphonomethyl) glycine) is a broad-spectrum biocide and is the active substance of the herbicide Roundup^®^. It was accidentally discovered by Henri Martin in 1950, however, it was in 1970 when Monsanto identified its herbicidal activity and formulated Roundup^®^ which has been commercially available since 1974 [5]. Although in the beginning glyphosate was used sparingly since its only application was as an aerosol spray, in 1996 genetically modified herbicide-tolerant plants accelerated its use.

Glyphosate and/or products containing it as an active substance have been suspected of causing several health problems over the years. Kidney and liver damage, various forms of cancer, and mental conditions such as Alzheimer’s and Parkinson’s disease have all been associated with the extensive use of glyphosate. Miscarriages, dermatological irritations, and respiratory problems have been reported after exposure to glyphosate. In addition, there are studies that have recorded an increase in infertility and malformations among pigs, as well as DNA damage and increased methylation in vitro [6].

Aside from glyphosate, Roundup^®^ contains polyethoxylated tallow amine (POEA), a surfactant that enhances the uptake and translocation of the active ingredient. However, Roundup^®^ contains many more ingredients for which the full list remains unknown, since it is considered a trade secret and is thus protected by the company. Consequentially, it has become more difficult to understand the risks posed by Roundup^®^ use [7]. Today, it is evident that the safety of Roundup^®^ is strongly doubted. Since 2015, over 300 lawsuits have been filed accusing the product of being detrimental to human health. Currently, several countries have banned its use, but its harmful effects are still present in the environment and in humans.

Bisphenol A

Bisphenol A (BPA) is an artificial compound that was first synthesized in 1891. It is used as a plasticizer in a wide variety of products such as food packaging, water bottles, thermal paper, and toys [8]. The most common use of BPA is for the manufacturing of epoxy resins that are used as an inner coating for metallic cans or other food or beverage packaging. BPA leaks out of epoxy resins and plastic bottles to contaminate food, beverages, and water [9]. The amount of BPA that leaks into stored foods and bottled water escalates when the plastics are exposed to high temperatures [8].

It has been proven that BPA causes endocrine disrupting effects in humans by interacting with nuclear receptors, such as estrogen receptors (ERs), androgen receptors (ARs), and thyroid hormone receptors (THRs). Since the endocrine system is affected, problems will automatically occur in the other systems of an organism. The reproductive system, the nervous system, metabolic function, immune function, and the growth and development of offspring are all adversely affected consequentially. Furthermore, associations between BPA levels and tumor development including prostate, breast, and lung cancer are added to the list of the effects of exposure to BPA [10].

Parabens

Parabens (PBs) are esters of para-hydroxybenzoic acid (pHBA) and were introduced to the market in 1930. They are used as antimicrobial agents and preservatives, and they have several applications in everyday products. In particular, they are used in deodorants, cosmetics, pharmaceuticals, and paper products such as baby wipes [11]. Moreover, PBs are the most common ingredient in personal care products, which makes them the two main sources of exposure. However, as expected, the environment is also burdened with PBs, mostly in aquatic environments, thereby creating a vicious cycle of exposure.

PBs have been blamed for exhibiting estrogenic activity, which is why an increasing amount of personal care products are currently being advertised as paraben-free. It has been found that parabens can disrupt the regular function of certain hormones such as androgens, estrogens, progesterone, and glucocorticosteroids by obstructing nuclear receptors [12]. They are also related to reproductive problems, breast cancer, obesity, genotoxicity, and allergies.

Authorities have set specific limitations regarding the use of PBs in personal care products and cosmetics. Since 2013, the European Commission has banned the use of specific parabens in all cosmetic and personal care products. In the current research, the toxicity of methyl (MePB), propyl (PrPB), and butyl (BuPB) parabens will be studied.

Triclosan

Triclosan (TCS) is a broad-spectrum antimicrobial agent that was first synthesized in the late 1960s. Initially, it was used as a disinfectant for hospitals and medical clinics but has since been used in soaps, toothpastes, cosmetics, and household cleaning products [13].

TCS has been blamed for disrupting the normal function of the endocrine system due to its structural similarity to estrogens. Moreover, it has the ability to bind to thyroid hormone receptors and has presented anti-androgenic and anti-estrogenic activity [14]. In addition, it is characterized as an allergen, one which either causes or enhances sensitivity to allergens.

In 2016, the FDA banned the use of TCS in soap, and in 2017 its usage was banned in all human hygiene biocidal products used within the European Union. Due to these bans, human exposure to TCS may be less than it was in previous years, however, there are still products that contain this substance. Another route for human exposure to TCS is the environment and especially the aquatic environment. Since this substance is present in the aquatic environment, water and natural products are being contaminated as well [13].

Bis (2-ethylhexyl) phthalate

DEHP is a phthalate ester used as a plasticizer in the production of polyvinyl chloride (PVC) to provide softer and more flexible materials. PVC containing DEHP is used in hospital equipment (intravenous tubing and bags), food wrapping, cable insulation, and automobile parts. DEHP is not bound to plastics with a covalent bond, and hence it may seep out of a product under certain conditions [15].

The main source of DEHP exposure for humans is in foodstuffs and bottled water, even though DEHP is not used in the manufacturing of water bottles. DEHP is more likely to leach into oily foods or foodstuffs that have a high concentration of lipids since it is highly soluble in oils but not very soluble in water. Other sources of exposure include house dust, indoor air, soil, or water sheds, but those are secondary sources only providing a minimal uptake [16].

After entering the body, DEHP is metabolized into several compounds including 2-ethyl hexanol, 2-ethyl hexanoic acid, and MEHP (mono (2-ethylhexyl) phthalate). Those metabolites are considered to be more toxic than the parent compound, especially MEHP, a substance that has been associated with antiandrogenic activity in humans [16]. DEHP itself also disrupts the normal function of the endocrine system. A decrease in aldosterone and testosterone concentrations after in utero exposure of male rats to DEHP has been reported in a previous study. This substance and its metabolites have also been demonstrated to affect some thyroid hormones in several animal models such as thyroxine (T4) and triiodothyronine (T3) in rats [15]. Considering the effects of DEHP in humans, children’s toys are not allowed to contain certain phthalates in the USA, Canada, and the European Union [15].

## 2. Study Design

### 2.1. Study Characteristics

This is a simulation study of human exposure to endocrine disruptors in real and daily life. The research is registered in the Animal Study Registry where further information such as the timeline and methods are available (DOI: 10.17590/asr.0000259). The protocol has been approved by the Committee of Ethics and Academic and Scientific Deontology of the University of Medicine and Pharmacy of Craiova (4/17 January 2020).

### 2.2. Study Group

Twenty rabbits (ten female and ten male), three months old and weighing about three kilos each, will be divided into five groups (four animals each) and placed in separate metal cages. After one week of the rabbits’ acclimatization, the administration of the compounds of interest will begin.

### 2.3. Sample Size Estimation

The number of animals as well as the methods of breeding and treatment should follow the rules of the 3Rs Principle (Refinement, Reduction, and Replacement) (https://nc3rs.org.uk/). These rules are sharp and strict to ensure the well-being of the animals as well as their restrained and rational use, and the Ethical Committee requires full compliance with these rules. To calculate the required sample size, a literature search was performed. According to the limited literature data, there is only one metanalysis supporting the glyphosate effect on the micronuclei of male mammalians, but there are no studied effects on rabbits’ micronuclei from glyphosate exposure. Due to the lack of corresponding scientific articles concerning the exposure of rabbits to the compounds of interest, data on the exposure of rats to the commercial form of glyphosate (Roundup^®^) were used [17,18,19]. The changes observed comparing the control group’s testosterone levels with the group exposed to Roundup^®^ ranged from 13.2 to 66.2%, depending on the dose administered. Based on the hypothesis of finding a testosterone change in rats and the accepted type I error of 5% and type II of 20%, it was estimated that four experimental animals per group were required to detect a percentage difference of 50%.

### 2.4. Breeding-Administration-Dosages

**Figure 1:**
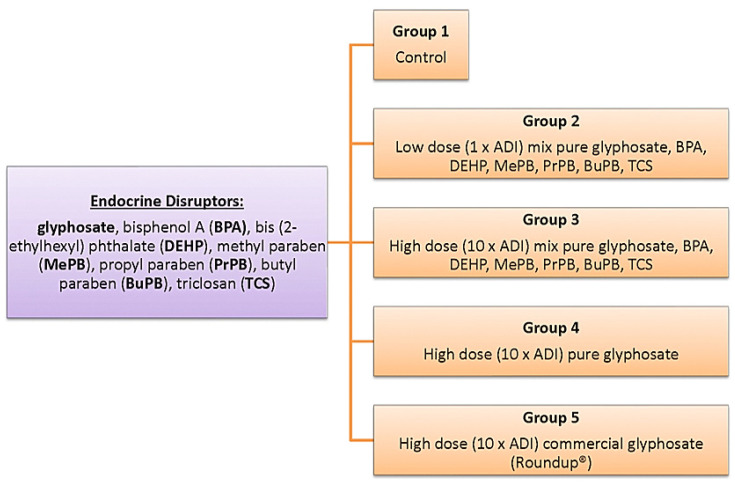
Groups of administration (substances-doses). ADI: Acceptable Daily Intake.

The first group will be the control group and will receive a normal diet (water, pellets, corn oil with 5% ethanol/water). In the second and third group, low and high doses of mixed BPA (≥99%), PBs (MePB, PrPB, BuPB) (HPLC) grade), TCS (100%), DEHP (PESTANAL^®^, analytical standard) and glyphosate (85%), will be administered while the fourth and fifth groups will receive a high dose of glyphosate in its pure and commercial form (Roundup^®^) (Figure 1). The substances will be dissolved in 5% ethanol/water to prepare the stock solutions. Specific amounts of each solution will be administered to the animals once a day, and mixed with their food five days per week for twelve consecutive months. The final daily amount of ethanol in the administered food will be less than 0.5%.

Dosages are based on the limits set by the European Food Safety Authority (EFSA) and the related service in Canada. According to the EFSA the ADI for BPA is set as 0.004 mg/kg body weight/day, for BuPB as 0.5 mg/kg body weight/day, for all PBs (MePB, PrPB) as 0–10 mg/kg body weight/day (considered 5 mg/kg body weight/day for MePB and 5 mg/kg body weight/day for PrPB), for total phthalates up to 0.05 mg/kg body weight/day, and for glyphosate up to 0.5 mg/kg body weight/day. The Government of Canada has set the ADI for TCS as 0.08 mg/kg body weight/day.

The low dose of the administered solution will contain an amount of each compound equal to its ADI (1× ADI), while the high dose will contain ten times the ADI (10× ADI).

All animals will be monitored and examined daily for signs of morbidity or mortality. Monitoring will include an examination of fur, mucous membranes, and secretions as well as an observation of animal behavior. Food and water consumption will also be recorded daily. Specifically, the consumption of the administration groups (groups 2, 3, 4, and 5) will be compared with the consumption of the control group (group 1) to identify any differences.

### 2.5. Sampling-Sacrifice

**Figure 2:**
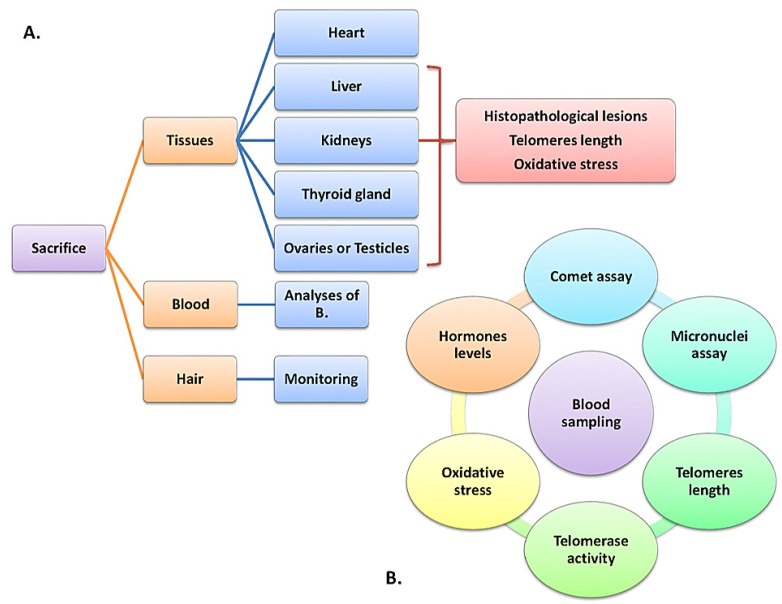
(**A**). Sacrifice procedure: obtained tissues and applied tests (**B**). Blood sampling and applied tests.

At the beginning of the in vivo experiment (t = 0 month) and every three months (t = 3, 6, 9 and 12 months), blood and hair samples will be collected and then analyzed to determine the levels of the compounds we are interested in (Figure 2).

Blood samples will also be used to assess a series of biomarkers that have been proved to be affected by exposure to substances with endocrine activity:

Micronuclei assay

Measurement of both the number of nucleated cells and the number of micro-nuclei in the dicotyledonous cells will be measured by testing the micro-nuclei in the lymphocytes. Cell culture, the addition of cytochalasin b (to stop cytokinesis while nuclear division carries on), and finally fixation of the cells are the main steps before measuring the micronuclei. By applying this method, we will study the toxicity of these substances in the genetic material of rabbits, using a method of high accuracy and a relatively low cost. Previous studies have already proven that exposure to glyphosate leads to greater rates of micronuclei formation [19].

Reproductive hormone levels

The substances that will be administered to the animals are characterized as endocrine disruptors which means that the endocrine system (controlling hormones) is the first to be affected. Thus, we will be able to determine the levels of the reproductive hormones progesterone, testosterone, androstenedione, and β-estradiol, but also of the thyroid hormones: thyroxine (T4), triiodothyronine (T3), and the thyroid stimulating hormone (T) using immunoassay techniques (e.g., Elisa kit).

Telomere length

Telomeres are repetitive sequences at the ends of chromosomes, which preserve chromosome integrity and offer protection from degradation, recombination, and fusion. Their length will be measured via Quantitative-Polymerase Chain Reaction (Q-PCR). After DNA extraction using an Absolute Human Telomere Length Quantification qPCR Assay Kit (ScienCell, USA), telomere length will be estimated by an automatic real-time PCR instrument. The attrition of telomeres after exposure to organic chemicals and pesticides has been recorded by Louzon and co-authors [20] rendering it worthy of investigation.

Telomerase activity

Telomerase is an enzyme responsible for the elongation of telomeres. Its activity will be measured via a Trap Elisa kit. Organic chemicals and pesticides are associated with a decrease in telomerase activity [20] and in this study we intend to verify this.

Oxidative stress

An assessment of oxidative stress will be accomplished by measuring the total antioxidant capacity (TAC), thiobarbituric acid reactive substances (TBARS), protein carbonyl concentrations, glutathione (GSH), glutathione peroxidase (GPx), glutathione reductase (GR), superoxide dismutase (SOD), and catalase quantities (CAT). Previously applied protocols [21,22,23,24,25] will be used and absorbance will be measured to determine redox biomarkers. Studies examining the associations between exposure to pesticides and oxidative stress biomarkers showed an increased oxidative stress [26] while the same was observed for endocrine disruptors mainly in mixtures, indicating a more profound effect compared to individual exposure [27].

Comet assay

DNA damage will be assessed using a single gel electrophoresis assay (SCGE). Lately, Kara and co-authors observed DNA damage and induced oxidative stress after exposure to pesticides [28].

All animals will be sacrificed at the end of the experiment (after twelve months of exposure) using phenobarbital sodium (Dolethal commercial solution, 5 mL/5 kg body weight), after first being anesthetized with a subcutaneous injection of Xylapan/Narketan solution (2/1) and will be necropsied. A complete macroscopic examination will be performed on the heart, liver, kidneys, brain, lungs, thyroid gland, and genitals.

After sacrifice, tissues and specifically kidneys, liver, heart, brain, lungs, thyroid gland, and genitals will be examined for histopathological lesions. At the same time, an oxidative stress assessment will be performed as well as a measurement of telomerase activity to determine the rate of its expression in the organs and the possible problems that may be associated with its reduced action.

## 3. Discussion

Studies in animals and humans have shown that health problems occur after systemic exposure to substances classified as endocrine disruptors. The literature so far reports the detection of the aforementioned substances in biological samples such as urine, blood, and hair [29,30,31,32], indicating the diffusion of the substances into the body and their possible accumulation in organs and/or adipose tissue. In the present study, the compounds of interest have been associated with neurological and behavioral problems; liver and kidney lesions; cancer mainly of the breast, skin, liver, or kidney; thyroid gland problems; adverse effects on the reproductive system, such as changes in the function and morphology of the ovaries and sperm motility; pregnancy problems; oxidative stress; increased chances of obesity; and allergies. The fact that there is evidence of possible genotoxicity of these substances, namely glyphosate, PBs, and BPA, also raises concerns [6,32,33,34,35].

Over the last few years, few studies have been conducted that simulate real life exposures to pesticides, food additives, and other chemicals found in consumer products (e.g., PBs). These in vivo studies have proved that exposures to such substances can lead to genotoxic and cytotoxic effects as well as changes in the redox profile of several tissues such as the liver, lungs, and brain [36,37]. Tsiaoussis and co-authors have concluded that being exposed to a wide variety of toxicants such as pesticides, aromatic hydrocarbons, and polychlorinated biphenyls highly affects the gut microbiome leading to metabolic, malignant, inflammatory, or immune system diseases [38]. These data raise doubts about the true extent of the burden these substances pose to people.

It is also worth noting that in terms of glyphosate, the environment—and consequently humans and animals—are not exposed in its pure form but in commercial forms. As it happens in all drugs and pesticides, except for the active substance, there are other additives that usually help to increase the stability and/or absorption of the drug by humans or plants, as in the case of pesticides [39]. These additives may act individually or synergistically with the active substance and have effects that are different or more pronounced than those of the active substance. In vivo and in vitro studies have shown that the commercial form of glyphosate has adverse effects on the animal bodies and cells compared to the pure form [40,41].

In the current study we aim to assess the effects of this kind of substance on several of the systems of an organism. The main point is that glyphosate, BPA, PBs, and TCS have been strongly associated with hormonal perturbation. Being the leading system of animal organisms, the hormonal system regulates the activity of all other systems. Hence, if hormonal function is changed all an organism’s systems are affected. Our main goal is to estimate the magnitude of these changes, when such substances act simultaneously, and their further health effects. The correlation of the implications of glyphosate in comparison with those of Roundup^®^ will also clarify the adverse synergistic effects of the additives.

## 4. Perspectives

This study aims to estimate the biological effects of long-term exposure to endocrine disruptors. The experimental protocol of this study has been designed based on the scarce literature data regarding rabbits and is restricted by the limitations of the 3Rs Principle. Until now, most researchers have focused on determining the associations and effects of exposure to one or two substances. However, people are exposed to the substances in question on a daily basis, unintentionally, and the real effects are still unknown. By studying the effects on the entire organism, the tissues, genetic material, the hormones, and the oxidative stress we will simulate real life exposure to these substances thus paving the way to unravelling the real health effects.
